# Duration of travel-associated faecal colonisation with ESBL-producing Enterobacteriaceae - A one year follow-up study

**DOI:** 10.1371/journal.pone.0205504

**Published:** 2018-10-24

**Authors:** Åse ÖstholmBalkhed, Maria Tärnberg, Maud Nilsson, Lennart E. Nilsson, Håkan Hanberger, Anita Hällgren, for the Southeast Sweden Travel Study Group

**Affiliations:** 1 Division of Infectious Disease, Department of Clinical and Experimental Medicine, Linköping University, Linköping, Sweden; 2 Department of Clinical and Experimental Medicine, Linköping University, Linköping, Sweden; Natural Environment Research Council, UNITED KINGDOM

## Abstract

**Background:**

In a previous study, we found that 30% of individuals travelling outside Scandinavia acquired extended-spectrum beta-lactamase-producing Enterobacteriaceae (ESBL-PE) in their faecal flora. The aim of this study was to determine the duration of travel-associated faecal colonisation with ESBL-PE, to assess risk factors for prolonged colonisation and to detect changes in antibiotic susceptibility during prolonged colonisation.

**Methods:**

Individuals with travel-associated colonisation with ESBL-PE submitted faecal samples every 3rd month over a one-year period. A questionnaire was completed at the beginning and end of follow-up. All specimens were analysed for ESBL-PE, and all isolates underwent confirmatory phenotype testing as well as molecular characterisation of ESBL-genes. Minimum inhibitory concentrations (MIC) for beta-lactam and non-beta-lactam agents were determined using the Etest.

**Results:**

Among 64 participants with travel-associated colonisation with ESBL-PE, sustained carriage was seen in 20/63 (32%), 16/63 (25%), 9/63 (14%) and 7/64 (11%) at 3, 6, 9 and 12 months after return from their journey, respectively. The majority, 44 (69%) of travellers were short-term carriers with ESBL-PE only detected in the initial post-travel stool sample. Evaluation of risk factors demonstrated a decreased risk of becoming a long-term carrier among travellers with diarrhoea while abroad and a history of a new journey during the follow-up period. High susceptible rates were demonstrated to carbapenems (97–100%), temocillin (95%), mecillinam (97%), amikacin (98%), fosfomycin (98%), nitrofurantoin (99%) and tigecycline (97%).

**Conclusion:**

Travel-associated faecal colonisation with ESBL-PE appears to be transient and generally brief. Diarrhoea while abroad or a new trip abroad during the follow-up period decreased the risk of becoming a long-term carrier. Only 11% of travellers who acquired ESBL-PE during their travels had sustained colonisation 12 months after return.

## Introduction

Antibiotic-resistant gram-negative bacteria, in particular extended-spectrum beta-lactamase (ESBL)-producing species, are becoming endemic in many parts of the world. Infections caused by ESBL-producing bacteria have increased dramatically over the last decade, and in parts of Asia, faecal carriage levels of more than 50% among asymptomatic individuals in the community have been reported [1–4]. However, there are still regions in the world, such as Scandinavia, where ESBL-PE is relatively rare [5].

Several studies have recognised international travel as a risk factor for acquisition of ESBL-producing Enterobacteriaceae (ESBL-PE) in the faecal flora [6–13]. Similarly, a number of studies have recognised travel as a risk factor for infections caused by ESBL-PE, in particular community-acquired urinary tract infections [14–16].

Our knowledge regarding factors that increase the risk for ESBL-PE infection has clinical implications, especially in the management of the critically ill. In the septic patient where the risk for ESBL-PE as the causal agent is high, the empiric antibiotic regimen should be broadened accordingly. This usually implies a shift from cephalosporins to carbapenems. Such a shift not only increases empiric coverage but also the risk for selection of carbapenemase-producing Enterobacteriaceae [17]. Targeted strategies designed to cope with the challenge of ESBL-PE are needed. Since a majority of infections are preceded by colonisation [18–20] knowledge of the duration of colonisation with ESBL-PE after travel could influence recommendations regarding management of gram-negative infections. In this respect, knowledge of the resistance profiles of travel-associated ESBL-PE and any change in resistance rates during prolonged colonisation, would also be of interest. However, duration of faecal colonisation with ESBL-PE after travel abroad has only been determined in a small number of studies with limited follow-up times or small numbers of individuals [6, 11, 21, 22].

In a previous study, we demonstrated that 68 of 226 (30%) Swedish travellers, previously not colonised with ESBL-PE, acquired ESBL-PE in their faecal flora while travelling outside Scandinavia [7]. The aim of the present study was to investigate the duration of travel-associated faecal colonisation with ESBL-PE, with focus on risk factors for prolonged colonisation, and change in antibiotic susceptibility during prolonged colonisation.

## Materials and methods

### Study design

The study design and methods have been described in detail elsewhere [7]. Briefly, individuals who acquired ESBL-PE in their faecal flora during travel outside Scandinavia were included in this multicentre, longitudinal, prospective cohort study. In the previous study, the participants submitted faecal samples and answered questionnaires providing demographic and medical background data as well as travel-associated data. Individuals with travel-associated (TA) colonisation with ESBL-PE were asked to provide faecal samples every third month over a one-year period after returning from their journey; in total one pre-travel sample, one post-travel sample and four follow-up samples. A final questionnaire was answered regarding antibiotic use and any further travel during the follow-up period. In cases where the respondent had another journey during the follow-up period, questions providing travel-associated data were answered. Self-collected faecal samples and questionnaires were sent to the clinical microbiology laboratory and study coordinators at Linköping University for analysis. In order to be eligible for final analyses, individuals were obliged to submit at least one faecal sample during the follow-up period and answer the final questionnaire in addition to providing the initial pre- and post-travel faecal samples and answering the first questionnaire.

Participants were defined as a short-term carrier if ESBL-PE was detected in the immediate post-travel sample only and no ESBL-PE found in subsequent samples. Long-term carriers were defined as individuals with isolates of ESBL-PE in one or more samples during the follow-up period after the immediate post-travel sample (i.e. duration ≥3 months). Duration of carriage was defined by the last positive sample harbouring ESBL-PE.

### Microbiological methods

Sample preparation, isolation of ESBL-PE, species identification and phenotypic ESBL-PE detection as well as susceptibility testing were performed using the same methods as in the previous study [7]. All phenotypically confirmed ESBL-PE isolates were examined for the presence of *bla*CTX-M [23]. Screening for genes belonging to the *bla*SHV and *bla*TEM families were limited to isolates where PCR was negative for *bla*CTX-M [24, 25]. Isolates not showing evidence of these three classical ESBL gene groups, were screened for the presence of *bla*AmpC according to a multiplex PCR analysis [26], as were isolates with an AmpC phenotype. All isolates from participants carrying ESBL and AmpC genes in different individual isolates from the same sample were examined for the presence of both genes.

Determination of minimal inhibitory concentrations (MICs) was performed using gradient testing with the Etest (BioMérieux, Marcy L’Etoile, France) according to the manufacturer’s instructions. *Escherichia coli* ATCC 25922 was used as a reference strain. MICs of beta-lactam agents (imipenem, meropenem, ertapenem, cefotaxime, ceftazidime, cefepime, piperacillin-tazobactam, amoxicillin-clavulanic acid, temocillin and mecillinam) and non-beta-lactam agents (amikacin, gentamicin, tobramycin, fosfomycin, trimethoprim-sulfamethoxazole, tigecycline, nitrofurantoin and ciprofloxacin) were determined. The European Committee on Antimicrobial Susceptibility Testing (EUCAST) clinical breakpoints were used to classify isolates as susceptible (S), intermediate (I) or resistant (R) [27]. For temocillin no breakpoint from EUCAST was available and a tentative breakpoint of 16/16 was used. The MIC of each drug was reported and MIC_50_ and MIC_90_ were calculated. Epidemiologic cut-offs, ECOFF, according to the EUCAST [28] were also used for imipenem and meropenem and isolates expressing MICs above ECOFF were subjected to whole genome sequencing for screening of resistance genes. Next generation sequencing was done using the Illumina MiSeq platform (Illumina, San Diego, CA, USA). Raw reads were assembled with CLC Genomics Workbench v.9.5.3 (Qiagen), and resistance genes were searched for using the ResFinder database (https://cge.cbs.dtu.dk/services/ResFinder/).

Multidrug-resistance was defined as decreased susceptibility (I or R) to a minimum of two antibiotics with different modes of action, in addition to the ESBL phenotype. We modified the definition of multidrug-resistance proposed by Magiorakos et al [29]; *i*) beta-lactamase inhibitors were represented by piperacillin-tazobactam and amoxicillin-clavulanic acid, *ii*) penicillins with possible activity against ESBL-PE were added and represented by temocillin and mecillinam, and *iii*) nitrofurantoin was added.

### Statistics

A logistic regression analysis was used to analyse risk factors for the persistence of ESBL-PE after travel, comparing short-term carriers with long-term carriers. The non-parametric Mann Whitney test was used to compare the crude MICs (1/2 MIC dilution steps as read on the Etest strip) of: *i*) immediate post-travel isolates vs. post-travel isolates taken at 3–12 months; *ii*) isolates from short-term carriers vs. those from long-term carriers; *iii*) immediate post-travel isolates from short-term carriers vs. immediate post-travel isolates from long-term carriers; and *iv)* immediate post-travel isolates from long-term carriers vs. post-travel isolates taken at 3–12 months. P-values <0.05 were considered statistically significant.

### Ethical considerations

The study was approved by the Regional Ethics Review Board in Linköping, Sweden (ref M94-08, T109-08). All participants provided written informed consent.

## Results

### Study population

We aimed to include all 68 patients with growth of ESBL-PE in the immediate post-travel sample, but 4 of these were excluded since they did not answer the final questionnaire, thus 64 participants (37 women and 27 men) were eligible for final analyses.

**Fig 1:**
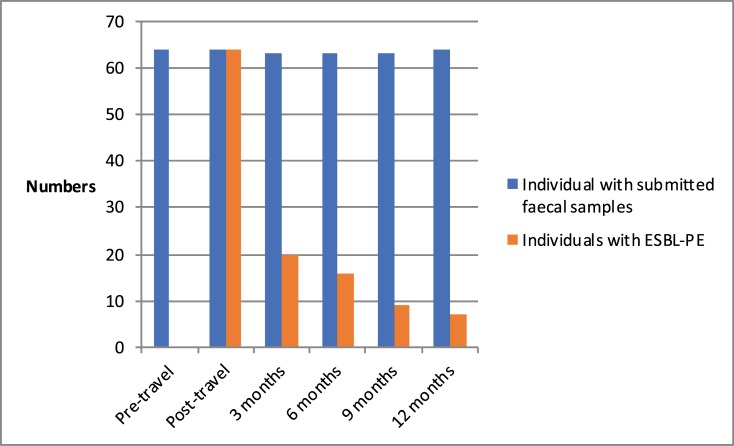
Dynamics of colonisation.

After the immediate post-travel sample, subsequent faecal samples were provided on four occasions at median times of 3 months (range -8d/+9d), 6 months and 3 days (range -6d/+15d), 9 months and 3 days (range -9d/+16d) and 12 months (range -7d/+12d) respectively. For simplicity the sampling times were subsequently referred to as immediate post-travel, and 3, 6, 9 and 12 months post-travel. At each sampling occasion, more than 98% of the participants provided the samples requested. Persistent and intermittent ESBL-PE carriage after acquisition was seen in 20/63 (32%), 16/63 (25%), 9/63 (14%) and 7/64 (11%) of participants at 3, 6, 9 and 12 months after the immediate post-travel sample, respectively (Fig 1).

Forty-four (69%) participants were short-term carriers, with ESBL-PE detected in the immediate post-travel sample only. Twenty (32%) participants were identified as long-term carriers with at least one ESBL-PE-positive faecal sample during the follow-up period. In two participants ESBL-PE was not found in the samples taken at 3 and 9 months, but was isolated from the samples following and these participants were consequently regarded as long-term carriers. Background data as well as travel-specific data for the short- and long-term carrier groups are presented in Table 1. Furthermore, Table 2 describes all events such as antibiotic treatment and new journeys, with or without travel-associated symptoms, during the follow-up period.

**Table 1:** Descriptive statistics of background and travel-associated data for short and long term carriers. ^#^Underlying comorbidities: diabetes, malignancy, inflammatory bowel disease (IBD), chronic urinary tract disease.

	Short-term carriers N = 41	Long-term carriers N = 23
**Background data**	**N (%)**	
Male	18 (43)	9 (39)
Female	23 (56)	14 (61)
Age (median)	54	55
<40	8 (20)	6 (26)
40–59	20 (49)	9 (39)
≥60	13 (32)	8 (35)
Any underlying comorbidity^#^	1 (2)	4 (17)
**Travel-associated data**		
Lenght of journey; days, median	15	16
Travel destination		
Europe	0	0
Africa, south of equator	11 (27)	2 (9)
Africa, north of equator	8 (20)	5 (22)
Asia (except Indian subcontinent)	15 (37)	10 (43)
Indian subcontinent	6 (15)	4 (17)
Australia and Oceania	0	0
South-america	2 (5)	2 (9)
North-america	0	0
Type of journey		
Visit to relatives and friends	4 (10)	5 (22)
Business journey	2 (5)	1 (4)
Tourist journey	28 (68)	16 (70)
“Backpacker-style”	8 (20)	3 (13)
Symptoms during journey		
Fever	5 (12)	2 (9)
Diarrhoea	26 (63)	9 (39)
Other gastrointestinal symptoms	14 (34)	4 (17)
Prophylaxis and treatment		
Antibiotic treatment during travel	2 (5)	3 (13)
Oral cholera vaccine before journey	22 (54)	13 (57)

**Table 2:** Events during the follow-up period for short and long term carriers.

	Short-term carriers, N = 41	Long-term carriers, N = 23
Events during follow-up period	N (%)	
Antibiotic treatment	9 (22)	4 (17)
New journey	33 (80)	11 (48)
Travel destination–new journey		
Europe	32 (78)	9 (39)
Africa, south of equator	0	1(4)
Africa, north of equator	3 (7)	1(4)
Asia (except Indian subcontinent)	6 (15)	2 (9)
Indian subcontinent	1 (2)	1(4)
Australia and Oceania	2 (5)	0
South America	1 (2)	1(4)
North America	4 (10)	0
Symptoms during journey		
Fever	1 (2)	0
Diarrhoea	4 (10)	1(4)
Other gastrointestinal symptoms	3 (7)	1(4)

## Risk factors for prolonged faecal colonisation

When comparing the two groups with respect to risk factors for prolonged colonisation, only two variables showed significance in the multivariable logistic regression model; diarrhoea during travel (OR = 0.26, p = 0.04) and a new journey during follow-up period (OR = 0.17, p = 0.01) both decreasing the risk of becoming a long-term-carrier. The benefit of having diarrhoea during travel on duration of colonization was most apparent among the oldest travelers, as none of the 8 individuals aged >60 y that were long-term carriers had diarrhoea during travel, whereas 10 of 13 of individuals aged > 60 y that were short-term carriers had diarrhoea during travel. There was a tendency towards prolonged colonisation among travellers with background comorbidity, but this was not significant in the multivariate analysis (Table 3). In S1 Table all data are provided.

**Table 3:** Risk factors for prolonged carrier state. Final multivariate logistic regression model after elimination of factors with p>0.15 in univariate analysis.

Variable	OR (95% CI)	P
New journey	0.17 (0.04–0.65)	0.01
Diarrhoea during first journey	0.26 (0.07–0.95)	0.04
Any background comorbidity	6.98 (0.57–85)	0.13

### Microbiological results

From samples taken from the 64 participants with TA colonisation, 171 isolates of ESBL-PE (165 *E*. *coli* and 6 *Klebsiella pneumoniae*) were detected. The microbial findings are further revealed in S2 Table.

All isolates with ESBL-producing *E*. *coli* were further analysed regarding MIC. MIC-distributions and susceptibility rates are presented in detail in S3 and S4 Tables. Overall susceptible rates to the cephalosporins (i.e. cefotaxime, ceftazidime and cefepime) were low; 4%, 13% and 17% respectively. All isolates were susceptible to meropenem. High susceptible rates were also seen for imipenem (99%) and ertapenem (97%). In all study participants, imipenem and meropenem clearly showed higher MICs in the 3-12-month post-travel samples when compared to the immediate post-travel samples (p <0.0001 and p = 0.0013, respectively). Susceptible rates for the beta lactam/beta-lactamase inhibitor combinations was lower than for the more ESBL stable penicillins which was high; amoxicillin-clavulanic acid (70%) < piperacillin-tazobactam (85%) < temocillin (95%) < mecillinam (97%). Among non-beta-lactam agents, high susceptible rates were observed for amikacin (98%), fosfomycin (98%), nitrofurantoin (99%) and tigecycline (97%). On the other hand, poor susceptible rates were seen to trimethoprim-sulfamethoxazole (32%), tobramycin (53%), gentamicin (61%) and ciprofloxacin (61%). Tobramycin showed lower MICs in the 3-12-month post-travel samples compared to the immediate post-travel samples from the long-term carriers (p = 0.0233). Gentamicin also expressed lower MICs in the 3-12-month post-travel samples compared to the immediate post-travel samples from the long-term carriers (p = 0.0407), as did nitrofurantoin (p = 0.0230). The susceptible rates for tobramycin and gentamicin in the immediate post-travel samples were 47% and 51%, respectively and in the faecal samples obtained 3–12 months post-travel the susceptible rates were 62% and 74% for these agents.

### Multidrug-resistance

Multidrug-resistance was detected in 107 (64%) isolates of ESBL-producing *E*. *coli* from 40 (63%) individuals. Of these, thirty-four (62%) isolates were from short-term carriers. In the immediate post-travel samples from long-term carriers, multidrug-resistance was demonstrated in 27 (66%) isolates. From faecal samples submitted 3 to 12 months after travel, 46 (67%) isolates of ESBL-producing *E*. *coli* were multidrug-resistant.

### Detection of ESBL-encoding genes

Among the *E*.*coli* isolates, ESBL-encoding genes were detected in 158 isolates; CTX-M was found in 131 isolates, SHV, TEM, and plasmid-mediated AmpC were found in 2, 3 and 23 isolates respectively. In 9 *E*. *coli* isolates with phenotypic ESBL, no corresponding ESBL- encoding genes were found. None of the six *K*. *pneumoniae* carried CTX-M. One isolate carried an undeterminable genotype. One isolate carried two inseparable SHV-alleles, and two isolates carried two inseparable SHV-alleles together with AmpC of DHA-type. Finally, two isolates carried SHV-alleles with ESBL-phenotype (SHV-2a and SHV-12, respectively).

Five *E*. *coli* isolates showed a MIC >0.125 mg/L for meropenem, and two of these showed a MIC >0.5 mg/L for imipenem. As this is considered a non-wild-type [28], the isolates were further characterised by whole genome sequencing where no carbapenemase-encoding genes were detected. In these isolates pAmpC (*bla*CMY-2) genes were found.

## Discussion

The main finding of this study was that colonisation with ESBL-PE after international travel generally appears to be transient. Only 11% of travellers who acquired ESBL-PE during their travel abroad had sustained colonisation 12 months after return which is a rate similar to that reported by Arcilla *et al*. [13]. One other important finding was that diarrhoea during travel or a new trip abroad during the follow-up period, decreased the risk of becoming a long-term carrier.

Our study supports the belief that travellers do not constitute a sustained reservoir of ESBL-PE in the community. However, the finding that 32% of travellers are colonised at least 3 months after returning from abroad, implies that the possibility of bacterial transmission to new hosts in both the community and the hospital setting must be taken into consideration. [30–33] The screening of all travellers for carriage of ESBL-PE upon return from abroad is neither feasible nor cost-effective. On the other hand, when prescribing a traveller returning from an endemic area, empirical antibiotic treatment for an infection that may be caused by a gram-negative agent, the risk of it being an ESBL-PE should be considered. Our data show that the risk decreases considerably with time, and that in most cases, colonisation in general does not last more than 6 months after return.

In this study, the rate of prolonged colonisation with ESBL-PE at 6 months was 25% which is in agreement with the study by Tängdén *et al*. [6], but higher than other similar studies [11, 13, 21, 22]. Whereas several studies have explored risk factors for acquisition of faecal of ESBL-PE during travel, studies addressing risk factors for prolonged colonisation with ESBL-PE after travel abroad are scarce.

Arcilla *et al*. as well as Ruppe *et al*. studied the impact of travel destination, duration of travel, species and CTX-M-type but results between these studies were contradictory [11, 13]. In this study we found no factors that prolonged colonization. However, we found that diarrhoea during travel (OR = 0.26, p = 0.04) or a new journey during the follow-up period (OR = 0.17, p = 0.01) both decreased the risk of becoming a long-term-carrier. Diarrhoea during travel has, by others [6, 10, 11, 21] and us [7], been found to be a risk factor for acquisition of ESBL-PE. It has been speculated that travellers’ diarrhoea lead to intestinal dysbiosis that decreases resistance to colonization by exogenous bacteria [34]. In this context, one might speculate that ESBL-PE, that manages to colonize without a preceding travellers’ diarrhoea-induced dysbiosis might be enriched in factors promoting colonization and thus be more prone to persist. Further studies are warranted on this subject. Similarly, no one has studied the effect of new travel on duration of colonization. Most of these second journeys were to low-prevalence countries within Europe. One might speculate that a new environment challenges the intestinal microbiota, hence clearing the newly acquired ESBL-PE, but further studies with larger number of individuals are needed to confirm these results.

*E*. *coli* isolates from post-travel samples at 3–12 months showed higher MICs for imipenem and meropenem. These isolates showed non-wild-type MICs, but were still under the EUCAST clinical breakpoint for resistance. No carbapenemase-encoding genes were detected in these isolates, but AmpC (*bla*CMY-2) genes were found. The non-wild type MICs is probably caused by this AmpC (*bla*CMY-2) in combination with overproduction of efflux pumps, and porin deficiency.

For non-beta-lactam agents such as tobramycin, gentamicin and nitrofurantoin the change in MICs were in the opposite direction, i.e. lower MICs in the samples obtained at 3–12 months post-travel. The reason for this shift in MICs remains unclear. For tobramycin and gentamicin, resistance rates in late post-travel samples were still high, 74% and 62% respectively, and these agents should not be considered for empirical treatment. A high rate of multidrug-resistance is common among ESBL-PE [21, 25, 35, 36] as was also the case in the isolates in this study. As a result, the therapeutic options in cases of post-travel clinical infection are limited.

The main limitation of this study is the lack of epidemiologic typing and that no investigation of phylogenetic groups was performed. Specific phylogenetic clonal lineages in ESBL-PE, such as the B2 phylogroup, sequence type (ST) 131 and the ST131 subclone H30-Rx have been linked to pandemic spread, prolonged carriage and increased potential to cause severe infections because of higher virulence [37–41]. Recent data have shown that ESBL-PE in community carriers in Sweden and healthy travelers are usually strains belonging to the non-B2 phylogroup, whereas healthcare-acquired strains are normally from the B2 phylogroup [12, 35, 42].

In conclusion, this study provides useful information regarding the transient nature of colonisation with ESBL-PE after travel abroad. However, 11% remain colonised one year after return from a journey abroad, and thus a history of travel in patients with bowel infection should always be obtained.

## Supporting information

S1 Table Risk factors. (XLS)

S2 Table Microbiological findings. (XLSX)

S3 Table MIC distributions of ESBL-producing *E*. *coli* isolates for beta lactam agents. Breakpoints according to EUCAST; S≤/R>. In absences of a EUCAST breakpoint for temocillin, a tentative breakpoint was used.
(DOCX)

S4 Table MIC distributions of ESBL-producing E. coli isolates for non-beta lactam agents. Breakpoints according to EUCAST; S≤/R>.
(DOCX)
