# Supplementary material for: Duration of travel-associated faecal colonisation with ESBL-producing Enterobacteriaceae - A one year follow-up study
Source: PLoS One. 2018 Oct 24;13(10):e0205504. doi: 10.1371/journal.pone.0205504 (PMC6200250; doi:10.1371/journal.pone.0205504)
Supplement: S3 Table — Breakpoints according to EUCAST; S≤/R>. In absences of a EUCAST breakpoint for temocillin, a tentative breakpoint was used. (DOCX) [file pone.0205504.s003.docx]

|  | Number of isolates with indicated MIC (mg/L) | | | | | | | | | | | | |  |  |  |
| --- | --- | --- | --- | --- | --- | --- | --- | --- | --- | --- | --- | --- | --- | --- | --- | --- |
|  | ≤ 0.032 | 0.064 | 0.125 | 0.25 | 0.5 | 1 | 2 | 4 | 8 | 16 | 32 | 64 | ≥ 128 | %S | %R | Breakpoints |
| Imipenem |  |  |  |  |  |  |  |  |  |  |  |  |  |  |  | 2/8 |
| All isolates, n=165 |  |  | 25 | **130** | 8 |  |  | 2 |  |  |  |  |  | 99 | 0 |  |
| immediate post-travel, n=96 |  |  | 22 | **74** |  |  |  |  |  |  |  |  |  | 100 | 0 |  |
| immediate post-travel, short time carriers, n=55 |  |  | 15 | **40** |  |  |  |  |  |  |  |  |  | 100 | 0 |  |
| immediate post-travel, long time carriers, n=42 |  |  | 7 | **34** |  |  |  |  |  |  |  |  |  | 100 | 0 |  |
| 3-12 months post-travel n=69 |  |  | 3 | **56** | 8 |  |  | 2 |  |  |  |  |  | 97 | 0 |  |
| Meropenem |  |  |  |  |  |  |  |  |  |  |  |  |  |  |  | 2/8 |
| All isolates | **147** | 12 | 1 | 3 |  | 1 | 1 |  |  |  |  |  |  | 100 | 0 |  |
| immediate post-travel | **90** | 6 |  |  |  |  |  |  |  |  |  |  |  | 100 | 0 |  |
| immediate post-travel, short time carriers | **51** | 4 |  |  |  |  |  |  |  |  |  |  |  | 100 | 0 |  |
| immediate post-travel, long time carriers | **39** | 2 |  |  |  |  |  |  |  |  |  |  |  | 100 | 0 |  |
| 3-12 months post-travel | **57** | 6 | 1 | 3 |  | 1 | 1 |  |  |  |  |  |  | 100 | 0 |  |
| Ertapenem |  |  |  |  |  |  |  |  |  |  |  |  |  |  |  | 0.5/1 |
| All isolates | **107** | 29 | 11 | 6 | 7 |  | 3 |  |  | 1 | 1 |  |  | 97 | 3 |  |
| immediate post-travel | **66** | 16 | 7 | 5 | 2 |  |  |  |  |  |  |  |  | 100 | 0 |  |
| immediate post-travel, short time carriers | **38** | 7 | 5 | 4 | 1 |  |  |  |  |  |  |  |  | 100 | 0 |  |
| immediate post-travel, long time carriers | **28** | 9 | 2 | 1 | 1 |  |  |  |  |  |  |  |  | 100 | 0 |  |
| 3-12 months post-travel | **41** | 13 | 4 | 1 | 5 |  | 3 |  |  | 1 | 1 |  |  | 93 | 7 |  |
| Cefotaxime |  |  |  |  |  |  |  |  |  |  |  |  |  |  |  | 1/2 |
| All isolates |  |  | 2 | 1 | 1 | 2 | 4 | 4 | 17 | 19 | 27 | **28** | 60 | 4 | 94 |  |
| immediate post-travel |  |  |  | 1 | 1 | 1 | 1 | 2 | 13 | 11 | 15 | **17** | 34 | 3 | 96 |  |
| immediate post-travel, short time carriers |  |  |  | 1 | 1 | 1 | 1 | 1 | 9 | 8 | **9** | 10 | 14 | 5 | 93 |  |
| immediate post-travel, long time carriers |  |  |  |  |  |  |  | 1 | 4 | 3 | 6 | **7** | 20 | 0 | 100 |  |
| 3-12 months post-travel |  |  | 2 |  |  | 1 | 3 | 2 | 4 | 8 | 12 | **11** | 26 | 4 | 91 |  |
| Ceftazidime |  |  |  |  |  |  |  |  |  |  |  |  |  |  |  | 1/4 |
| All isolates |  |  |  |  | 8 | 14 | 28 | 24 | **20** | 35 | 17 | 5 | 14 | 13 | 55 |  |
| immediate post-travel |  |  |  |  | 4 | 10 | 16 | 11 | **9** | 23 | 15 | 3 | 5 | 15 | 57 |  |
| immediate post-travel, short time carriers |  |  |  |  | 3 | 6 | 10 | 4 | **7** | 10 | 10 | 2 | 3 | 16 | 58 |  |
| immediate post-travel, long time carriers |  |  |  |  | 1 | 4 | 6 | 7 | 2 | **13** | 5 | 1 | 2 | 12 | 56 |  |
| 3-12 months post-travel |  |  |  |  | 4 | 4 | 12 | 13 | **11** | 12 | 2 | 2 | 9 | 12 | 52 |  |
| Cefepime |  |  |  |  |  |  |  |  |  |  |  |  |  |  |  | 1/4 |
| All isolates |  |  | 2 | 7 | 6 | 13 | 25 | **37** | 34 | 26 | 9 | 3 | 3 | 17 | 45 |  |
| immediate post-travel |  |  | 2 | 6 | 2 | 7 | 13 | **23** | 16 | 18 | 6 | 2 | 1 | 18 | 44 |  |
| immediate post-travel, short time carriers |  |  | 2 | 5 | 2 | 4 | 8 | **15** | 9 | 5 | 3 | 1 | 1 | 24 | 35 |  |
| immediate post-travel, long time carriers |  |  |  | 1 |  | 3 | 5 | 8 | **7** | 13 | 3 | 1 |  | 10 | 59 |  |
| 3-12 months post-travel |  |  |  | 1 | 4 | 6 | 12 | **14** | 18 | 8 | 3 | 1 | 2 | 16 | 46 |  |
| Piperacillin-tazobactam |  |  |  |  |  |  |  |  |  |  |  |  |  |  |  | 8/16 |
| All isolates |  |  |  |  | 1 | 14 | **78** | 35 | 13 | 6 | 1 |  | 17 | 85 | 11 |  |
| immediate post-travel |  |  |  |  | 1 | 10 | **43** | 21 | 12 | 3 | 1 |  | 5 | 91 | 6 |  |
| immediate post-travel, short time carriers |  |  |  |  | 1 | 5 | **24** | 12 | 6 | 3 |  |  | 4 | 87 | 7 |  |
| immediate post-travel, long time carriers |  |  |  |  |  | 5 | **19** | 9 | 6 |  | 1 |  | 1 | 95 | 5 |  |
| 3-12 months post-travel |  |  |  |  |  | 4 | **35** | 14 | 1 | 3 |  |  | 12 | 78 | 17 |  |
| Amoxicillin-clavulanic acid |  |  |  |  |  |  |  |  |  |  |  |  |  |  |  | 8/8 |
| All isolates |  |  |  |  |  |  |  | 28 | **87** | 28 | 11 | 3 | 8 | 70 | 30 |  |
| immediate post-travel |  |  |  |  |  |  |  | 21 | **54** | 16 | 5 |  |  | 78 | 22 |  |
| immediate post-travel, short time carriers |  |  |  |  |  |  |  | 13 | **30** | 9 | 3 |  |  | 78 | 22 |  |
| immediate post-travel, long time carriers |  |  |  |  |  |  |  | 8 | **24** | 7 | 2 |  |  | 78 | 22 |  |
| 3-12 months post-travel |  |  |  |  |  |  |  | 7 | **33** | 12 | 6 | 3 | 8 | 58 | 42 |  |
| Temocillin |  |  |  |  |  |  |  |  |  |  |  |  |  |  |  | 16/16 |
| All isolates |  |  |  |  |  |  | 4 | 33 | **89** | 31 | 8 |  |  | 95 | 5 |  |
| immediate post-travel |  |  |  |  |  |  | 4 | 22 | **47** | 19 | 4 |  |  | 96 | 4 |  |
| immediate post-travel, short time carriers |  |  |  |  |  |  | 2 | 14 | **23** | 14 | 2 |  |  | 96 | 4 |  |
| immediate post-travel, long time carriers |  |  |  |  |  |  | 2 | 8 | **24** | 5 | 2 |  |  | 95 | 5 |  |
| 3-12 months post-travel |  |  |  |  |  |  |  | 11 | **42** | 12 | 4 |  |  | 94 | 6 |  |
| Mecillinam |  |  |  |  |  |  |  |  |  |  |  |  |  |  |  | 8/8 |
| All isolates |  | 1 | 9 | 27 | **53** | 32 | 22 | 12 | 4 | 2 | 1 |  | 2 | 97 | 3 |  |
| immediate post-travel |  | 1 | 7 | 18 | **30** | 18 | 12 | 6 | 2 |  | 1 |  | 1 | 98 | 2 |  |
| immediate post-travel, short time carriers |  | 1 | 5 | 11 | **17** | 7 | 8 | 3 | 1 |  | 1 |  | 1 | 96 | 4 |  |
| immediate post-travel, long time carriers |  |  | 2 | 7 | **13** | 11 | 4 | 3 | 1 |  |  |  |  | 100 | 0 |  |
| 3-12 months post-travel |  |  | 2 | 9 | 23 | **14** | 10 | 6 | 2 | 2 |  |  | 1 | 96 | 4 |  |
