# Supplementary material for: Duration of travel-associated faecal colonisation with ESBL-producing Enterobacteriaceae - A one year follow-up study
Source: PLoS One. 2018 Oct 24;13(10):e0205504. doi: 10.1371/journal.pone.0205504 (PMC6200250; doi:10.1371/journal.pone.0205504)
Supplement: S4 Table — Breakpoints according to EUCAST; S≤/R>. (DOCX) [file pone.0205504.s004.docx]

|  | Number of isolates with indicated MIC (mg/L) | | | | | | | | | | | | |  |  |  |
| --- | --- | --- | --- | --- | --- | --- | --- | --- | --- | --- | --- | --- | --- | --- | --- | --- |
|  | ≤0.032 | 0.064 | 0.125 | 0.25 | 0.5 | 1 | 2 | 4 | 8 | 16 | 32 | 64 | ≥128 | %S | %R | Breakpoints |
| Amikacin |  |  |  |  |  |  |  |  |  |  |  |  |  |  |  | 8/16 |
| All isolates |  |  |  |  |  | 12 | **124** | 23 | 3 | 2 | 1 |  |  | 98 | 1 |  |
| immediate post-travel |  |  |  |  |  | 10 | **70** | 12 | 1 | 2 | 1 |  |  | 97 | 1 |  |
| immediate post-travel, short time carriers |  |  |  |  |  | 8 | **39** | 5 |  | 2 | 1 |  |  | 95 | 2 |  |
| immediate post-travel, long time carriers |  |  |  |  |  | 2 | **31** | 7 | 1 |  |  |  |  | 100 | 0 |  |
| 3-12 months post-travel |  |  |  |  |  | 2 | **54** | 11 | 2 |  |  |  |  | 100 | 0 |  |
| Gentamicin |  |  |  |  |  |  |  |  |  |  |  |  |  |  |  | 2/4 |
| All isolates |  |  | 9 | 71 | **17** | 3 |  | 2 | 5 | 19 | 26 | 7 | 6 | 61 | 38 |  |
| immediate post-travel |  |  | 5 | 40 | 3 | **1** |  | 2 | 4 | 15 | 19 | 4 | 3 | 51 | 47 |  |
| immediate post-travel, short time carriers |  |  | 4 | 23 | **2** |  |  | 1 | 2 | 12 | 7 | 3 | 1 | 53 | 45 |  |
| immediate post-travel, long time carriers |  |  | 1 | 17 | 1 | 1 |  | **1** | 2 | 3 | 12 | 1 | 2 | 49 | 49 |  |
| 3-12 months post-travel |  |  | 4 | **31** | 14 | 2 |  |  | 1 | 4 | 7 | 3 | 3 | 74 | 26 |  |
| Tobramycin |  |  |  |  |  |  |  |  |  |  |  |  |  |  |  | 2/4 |
| All isolates |  |  |  |  | 45 | **40** | 3 | 2 | 31 | 28 | 10 | 6 |  | 53 | 45 |  |
| immediate post-travel |  |  |  |  | 19 | 24 | 2 | 1 | **20** | 17 | 8 | 5 |  | 47 | 52 |  |
| immediate post-travel, short time carriers |  |  |  |  | 11 | **17** |  |  | 9 | 10 | 5 | 3 |  | 51 | 49 |  |
| immediate post-travel, long time carriers |  |  |  |  | 8 | 7 | 2 | 1 | **11** | 7 | 3 | 2 |  | 41 | 56 |  |
| 3-12 months post-travel |  |  |  |  | 26 | **16** | 1 | 1 | 11 | 11 | 2 | 1 |  | 62 | 36 |  |
| Fosfomycin |  |  |  |  |  |  |  |  |  |  |  |  |  |  |  | 32/32 |
| All isolates |  | 4 | 17 | **87** | 40 | 8 | 3 |  |  |  | 2 | 1 | 3 | 98 | 2 |  |
| immediate post-travel |  | 1 | 12 | **51** | 24 | 1 | 2 |  |  |  | 2 |  | 3 | 97 | 3 |  |
| immediate post-travel, short time carriers |  |  | 6 | **30** | 13 | 1 | 2 |  |  |  | 2 |  | 1 | 98 | 2 |  |
| immediate post-travel, long time carriers |  | 1 | 6 | **21** | 11 |  |  |  |  |  |  |  | 2 | 95 | 5 |  |
| 3-12 months post-travel |  | 3 | 5 | **36** | 16 | 7 | 1 |  |  |  |  | 1 |  | 99 | 1 |  |
| Trimethoprim-sulfamethoxazole |  |  |  |  |  |  |  |  |  |  |  |  |  |  |  | 2/4 |
| All isolates | 11 | 21 | 8 | 4 | 4 | 1 | 3 | 1 | 1 | 1 | **110** |  |  | 32 | 68 |  |
| immediate post-travel | 5 | 11 | 6 | 2 | 3 |  |  |  |  |  | **69** |  |  | 28 | 72 |  |
| immediate post-travel, short time carriers | 3 | 7 | 3 | 1 | 1 |  |  |  |  |  | **40** |  |  | 27 | 73 |  |
| immediate post-travel, long time carriers | 2 | 4 | 3 | 1 | 2 |  |  |  |  |  | **29** |  |  | 29 | 71 |  |
| 3-12 months post-travel | 6 | 10 | 2 | 2 | 1 | 1 | 3 | 1 | 1 | 1 | **41** |  |  | 36 | 64 |  |
| Tigecycline |  |  |  |  |  |  |  |  |  |  |  |  |  |  |  | 1/2 |
| All isolates |  |  |  | 34 | **97** | 29 | 5 |  |  |  |  |  |  | 97 | 0 |  |
| immediate post-travel |  |  |  | 17 | **60** | 17 | 2 |  |  |  |  |  |  | 98 | 0 |  |
| immediate post-travel, short time carriers |  |  |  | 9 | **35** | 9 | 2 |  |  |  |  |  |  | 96 | 0 |  |
| immediate post-travel, long time carriers |  |  |  | 8 | **25** | 8 |  |  |  |  |  |  |  | 100 | 0 |  |
| 3-12 months post-travel |  |  |  | 17 | **37** | 12 | 3 |  |  |  |  |  |  | 96 | 0 |  |
| Nitrofurantoin |  |  |  |  |  |  |  |  |  |  |  |  |  |  |  | 64/64 |
| All isolates |  |  |  |  |  |  | 1 | 15 | 52 | **53** | 36 | 6 | 2 | 99 | 1 |  |
| immediate post-travel |  |  |  |  |  |  | 1 | 11 | 33 | **24** | 20 | 5 | 2 | 98 | 2 |  |
| immediate post-travel, short time carriers |  |  |  |  |  |  | 1 | 6 | 16 | **12** | 14 | 5 | 1 | 98 | 2 |  |
| immediate post-travel, long time carriers |  |  |  |  |  |  |  | 5 | **17** | 12 | 6 |  | 1 | 98 | 2 |  |
| 3-12 months post-travel |  |  |  |  |  |  |  | 4 | 19 | **29** | 16 | 1 |  | 100 | 0 |  |
| Ciprofloxacin |  |  |  |  |  |  |  |  |  |  |  |  |  |  |  | 0.25/0.5 |
| All isolates | 65 | 6 | 3 | **27** | 6 | 3 | 1 |  | 1 | 2 | 51 |  |  | 61 | 35 |  |
| immediate post-travel | 33 | 4 | 3 | **18** | 4 | 3 | 1 |  | 1 | 2 | 27 |  |  | 60 | 35 |  |
| immediate post-travel, short time carriers | 23 | 2 | 1 | **9** | 2 | 2 | 1 |  |  | 1 | 14 |  |  | 64 | 33 |  |
| immediate post-travel, long time carriers | 10 | 2 | 2 | **9** | 2 | 1 |  |  | 1 | 1 | 13 |  |  | 56 | 39 |  |
| 3-12 months post-travel | 32 | 2 |  | **9** | 2 |  |  |  |  |  | 24 |  |  | 62 | 35 |  |
